# Supplementary material for: High sensitivity variable-temperature infrared nanoscopy of conducting oxide interfaces
Source: Nat Commun. 2019 Jun 24;10:2774. doi: 10.1038/s41467-019-10672-5 (PMC6591405; doi:10.1038/s41467-019-10672-5)
Supplement: Supplementary file 1 — Supplementary Information [file 41467_2019_10672_MOESM1_ESM.pdf]

Supplementary Information: High sensitivity variable-temperature infrared nanoscopy of conducting oxide interfaces

Weiwei Luo,* Margherita Boselli,* Jean-Marie Poumirol, Ivan Ardizzone, Jérémie Teyssier, Dirk van der Marel, Stefano Gariglio, Jean-Marc Triscone, and Alexey B. Kuzmenko†

Department of Quantum Matter Physics, University of Geneva, Quai Ernest-Ansermet 24, 1211 Geneva, Switzerland

* Contributed equally to this work

† Alexey.Kuzmenko@unige.ch

Supplementary Note 1: Modelling the optical dielectric functions of LAO, STO and 2DES

In our simulations, we parameterized the complex dielectric functions of insulating $SrTiO_3$ and $LaAlO_3$ using the Lorentz dispersion model:

$$\epsilon = \epsilon_\infty + \sum_{j=1} \frac{\omega_{\mathrm{p}j}^2}{\omega_{0j}^2 - \omega^2 - \mathrm{i}\gamma_j \omega}. \tag{1}$$

Here ϵ_∞ is the high frequency dielectric constant. The parameters $\omega_{\mathrm{p}j}$, ω_{0j} and γ_j are the plasma frequency, the resonant frequency and the scattering rate respectively of the j-th Lorentz oscillator describing an individual optical phonon. We adopted the parameters of LAO from Ref.[1] and the parameters of STO from Ref.[2]. The actual values for both compounds used in our calculations are presented in Supplementary Table 1 and Supplementary Table 2.

Supplementary Table 1. Model Lorentz parameters for STO (ϵ_∞=5.37)

j	ω_{0j} (cm^{-1})	$\omega_{\mathrm{p}j}$ (cm^{-1})	γ_j (cm^{-1})
1	92.0	1587.5	9.8
2	175.4	332.4	3.1
3	539.1	617.6	25.9

Supplementary Table 2. Model Lorentz parameters for LAO (ϵ_∞=4)

j	ω_{0j} (cm^{-1})	$\omega_{\mathrm{p}j}$ (cm^{-1})	γ_j (cm^{-1})
1	184.0	698.2	4.2
2	428.0	866.6	2.7
3	496.0	80.0	16.0
4	652.0	338.8	23.5
5	692.0	113.7	32.0

To model the dielectric function of the 2DES, we add a Drude term to the dielectic

function of the insulating STO[3, 4]:

$$\epsilon_{\rm 2DES} = \epsilon_{\rm STO} - \frac{n_{\rm 3D}e^2}{m^*\epsilon_0(\omega^2 + {\rm i}\omega\tau^{-1})}. \quad (2)$$

An exponential distribution of $n_{\rm 3D}$ from the interface is considered:

$$n_{\rm 3D}(z) = n_{\rm 2D}/z_0 \cdot \exp(-z/z_0), \quad (3)$$

where $n_{\rm 2D}$ is the two dimensional carrier density, z is the distance from the interface, and z_0 is the decay length.

In the calculations, the exponential distribution of $n_{\rm 3D}$ is simulated by dividing the 2DES layer (having the thickness $z_{\rm max}$ defined below) into a large number $N_{\rm d}$ of layers with a constant value of $n_{\rm 3D}$, for which the thickness are chosen in order to achieve an even distribution of $n_{\rm 3D}$ from $n_{\rm 3D}(z=0) = n_{\rm 2D}/z_0$ to $n_{\rm 3D}(z_{\rm max}) = n_{\rm 2D}/z_0 \cdot r_{\rm min}$, where $r_{\rm min}$ is a specially chosen small parameter. Specifically, we use $N_{\rm d} = 100$, and $r_{\rm min} = 1 \times 10^{-3}$. We checked that if $N_{\rm d}$ is further increased or $r_{\rm min}$ is further decreased then the calculation results change insignificantly.

Supplementary Note 2: The influence of amorphous-STO

In order to estimate the effect of the amorphous STO, we measured the near-field signal amplitude and phase on a sample with a reference region covered by a-STO but without LAO (see Supplementary Fig. 1). The near-field amplitude of c-STO is 93% of that measured on a-STO and there is no measurable phase contrast. The small amplitude contrast is caused either by some difference between the optical dielectric functions of c-STO and a-STO or by an additional light scattering on the amorphous STO.

Supplementary Note 3: Calculation of $r_{\rm p}(q,\omega)$

To calculate the reflection coefficient, we consider a system of n layers ($j = 2, 3, ...n+1$ from top to bottom) between the dielectric environment ($j = 1$, in our case vacuum, with $\epsilon_1 = 1$) and substrate (s, in our case STO with the dielectric function $\epsilon_{\rm s} = \epsilon_{\rm STO}$). The dielectric function and thickness are ϵ_j and d_j respectively for the j ($j = 2, 3, ...n+1$) layer. For a given in-plane wavevector q, the out-of-plane wavevectors in the j ($j = 1, 2, ...n+1$)

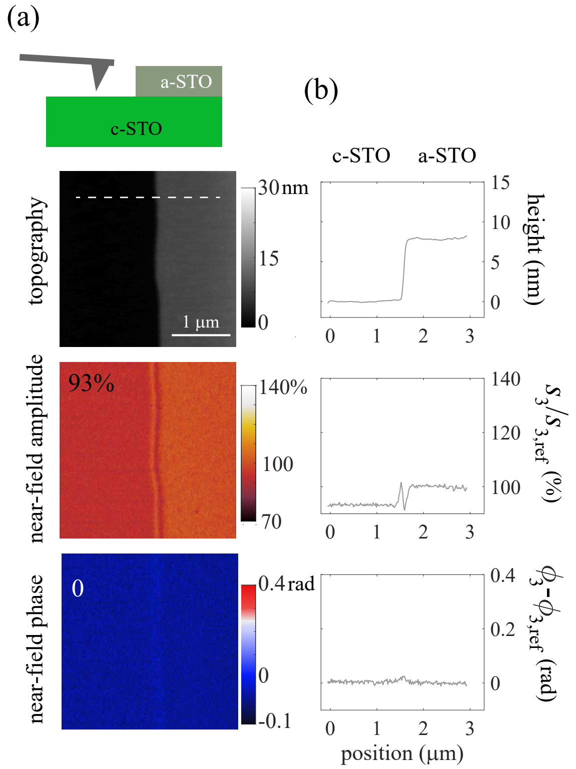

Supplementary Figure 1. (a) Sample description, AFM height topography, near-field amplitude (s_3) and near-field phase (ϕ_3) at room temperature for the sample of a-STO/c-STO without LAO. (b) AFM height, near-field amplitude and phase profiles along the dashed line in (a). Near-field signal is normalized with respect to that of a-STO. The laser wavelength is 10.7 μm.

dielectric and the substrate are $k_j = \sqrt{\epsilon_j q_0^2 - q^2}$ and $k_\mathrm{s} = \sqrt{\epsilon_\mathrm{s} q_0^2 - q^2}$, respectively, where $q_0 = \omega/c$ is the vacuum wavevector.

Within the transfer matrix method[5–7], the reflection coefficient can be calculated recurrently from the bottom to the top layer. For $j = n, n-1, ...1$, the reflection coefficient $r_{j,\mathrm{s}}$ between the j dielectric and substrate is

$$r_{j,\mathrm{s}} = \frac{r_{j,j+1} + r_{j+1,\mathrm{s}}\tau_{j+1}^2}{1 + r_{j,j+1}r_{j+1,\mathrm{s}}\tau_{j+1}^2}, \tag{4}$$

where

$$r_{j,j+1} = \frac{k_j\epsilon_{j+1} - k_{j+1}\epsilon_j}{k_j\epsilon_{j+1} + k_{j+1}\epsilon_j}, \tag{5}$$

$$r_{n+1,\mathrm{s}} = \frac{k_{n+1}\epsilon_\mathrm{s} - k_\mathrm{s}\epsilon_{n+1}}{k_{n+1}\epsilon_\mathrm{s} + k_\mathrm{s}\epsilon_{n+1}} \tag{6}$$

and $\tau_j = e^{\mathrm{i}k_j d_j}$.

Supplementary Note 4: The point dipole model

In the point dipole model[8, 9], the tip is approximated by a point dipole at its apex, with the polarizability $\sim a^3$, where a is the radius of the tip. The cantilever resonates at a tapping frequency Ω, therefore the distance between the tip and the sample is:

$$z_{\rm d}(t) = b + \Delta z(1 - \cos\Omega t), \tag{7}$$

where b is the minimum tip-sample distance and Δz =60 nm is the tapping amplitude.

In the s-SNOM experiment, the p-polarized incident light is used. The tip-sample coupling function G is obtained by integrating over all in-plane momenta:

$$G(z_{\rm d}, \omega) = \int_0^\infty dq\, q^2 e^{-2qz_{\rm d}} r_{\rm p}(q, \omega). \tag{8}$$

The weight function $q^2 e^{-2qz_{\rm d}}$ is time-dependent. The time averaged $\langle q^2 e^{-2qz_{\rm d}} \rangle_t$ has a maximum around $q = 1.38 \times 10^5$ cm^{-1}, by considering a minimum tip-sample distance $b = 50$ nm.

The demodulated n-th harmonics of the near-field signal is

$$s_n {\rm e}^{{\rm i}\phi_n} \propto \int_0^{2\pi} \frac{{\rm e}^{{\rm i}n\phi} d\phi}{1 - G(b + \Delta z(1 - \cos\phi), \omega) a^3}. \tag{9}$$

Supplementary Note 5: Near-field images at different temperatures

In Supplementary Fig. 2a we present the temperature dependence of the DC resistance of the LAO(5 u.c.)/2DES/STO sample. The topography and s-SNOM images at different temperatures on this sample are shown in Supplementary Fig. 2b and c. An interesting observation is the presence of a weak stripe pattern in the near-field maps. It is clearly related to the atomic terrace steps in STO as is evidenced from the AFM topography image of the same area. This further demonstrates the potential of s-SNOM for imaging nanoscale spatial modulation of the 2DES properties.

Supplementary Note 6: Influence of tip radius a, minimum distance b , carrier density $n_{\rm 2D}$ and decay length z_0 on calculated near-field signals

In the main text, we use a=50 nm, b=50 nm, $n_{\rm 2D}$=8×10^{13} cm^{-2} and z_0=2 nm. In Supplementary Fig. 3 and Supplementary Fig. 4 we demonstrate how a reasonable variation

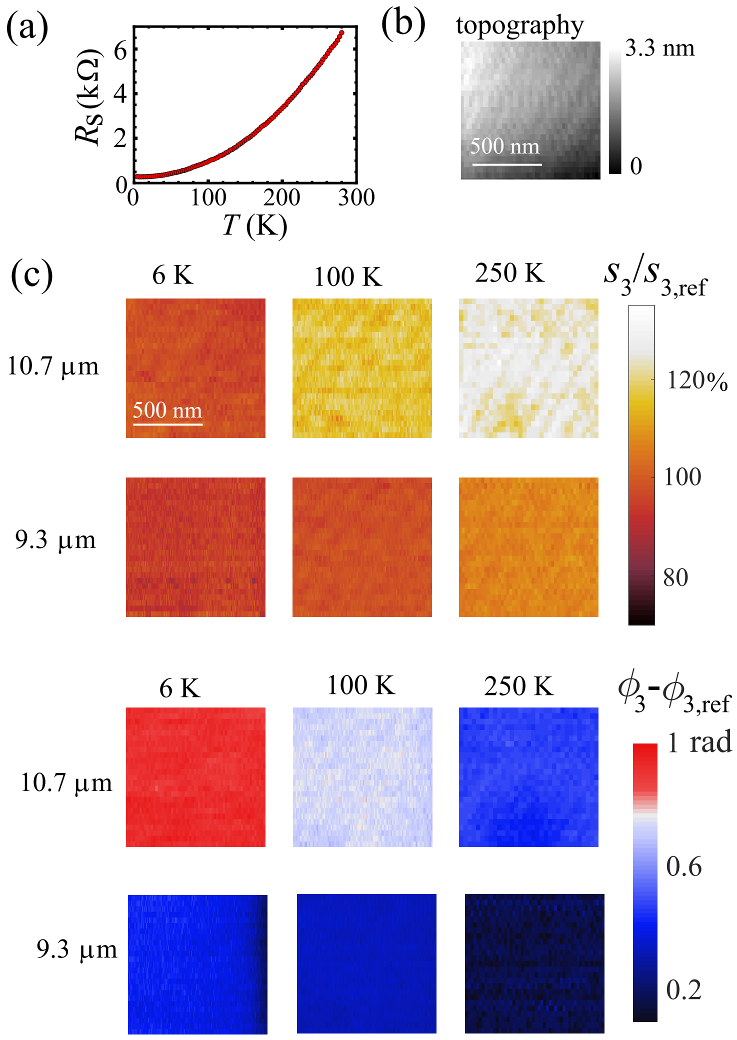

Supplementary Figure 2. (a) Temperature dependent resistance of the sample LAO(5 u.c.)/2DES/STO. (b) Topography of a 1 × 1 µm area on LAO/c-STO taken at 250 K. (c) Near-field amplitude and phase images of the same area at 6 K, 100 K and 250 K, for two laser wavelengths: 10.7 µm and 9.3 µm. All the images are normalized with respect to those on LAO/a-STO.

of these parameters affects the calculation shown in Figure 3b of the main text. Namely, in Supplementary Fig. 3 the near-field signals as a function of the optical mobility are plotted for different values of a and b. In Supplementary Fig. 4 the same quantities are calculated for other values of n_{2D} and z_0.

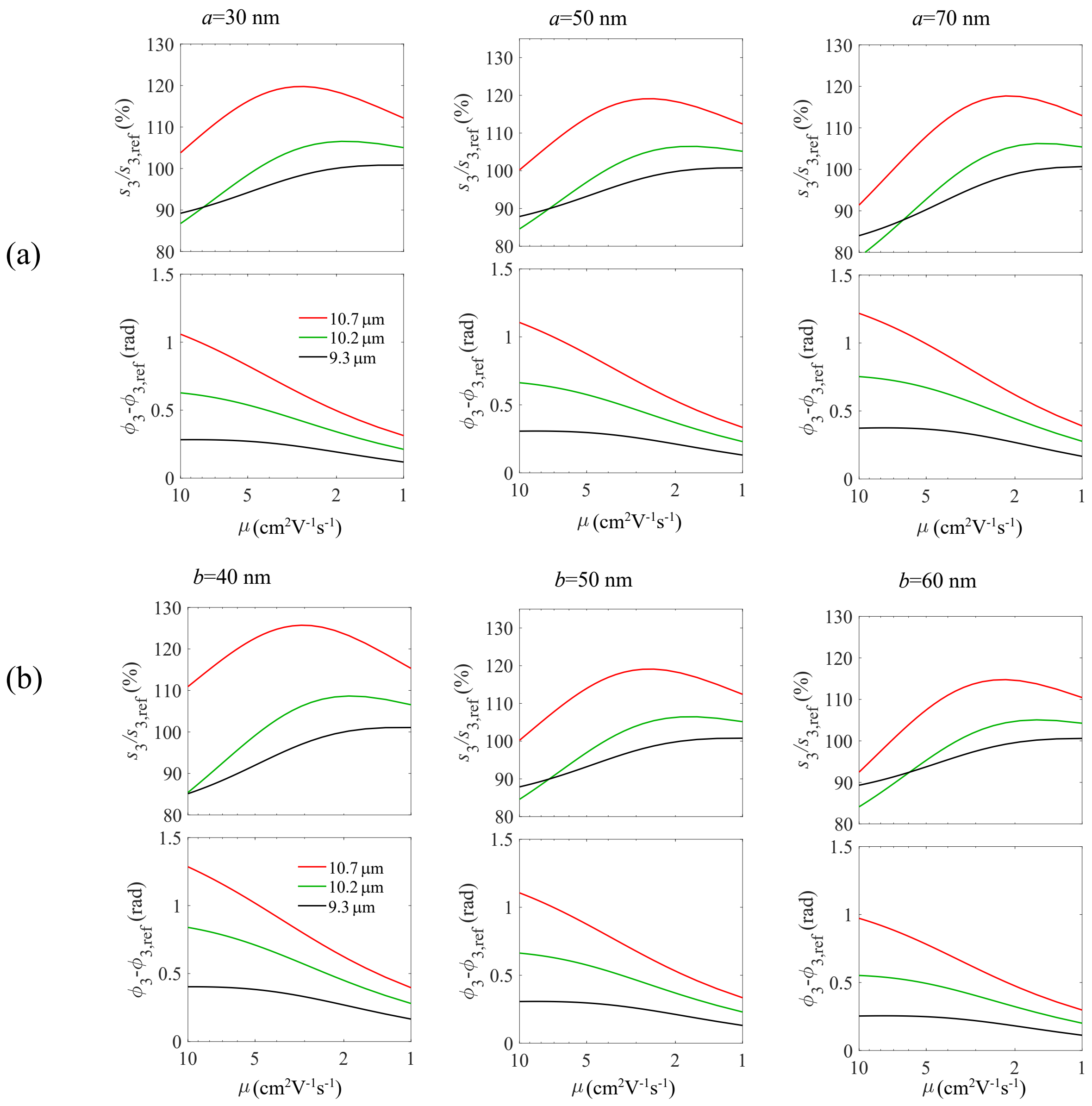

Supplementary Figure 3. Influence of the tip radius a (a) and the tip-sample minimum distance b (b) on the calculated near-field contrasts.

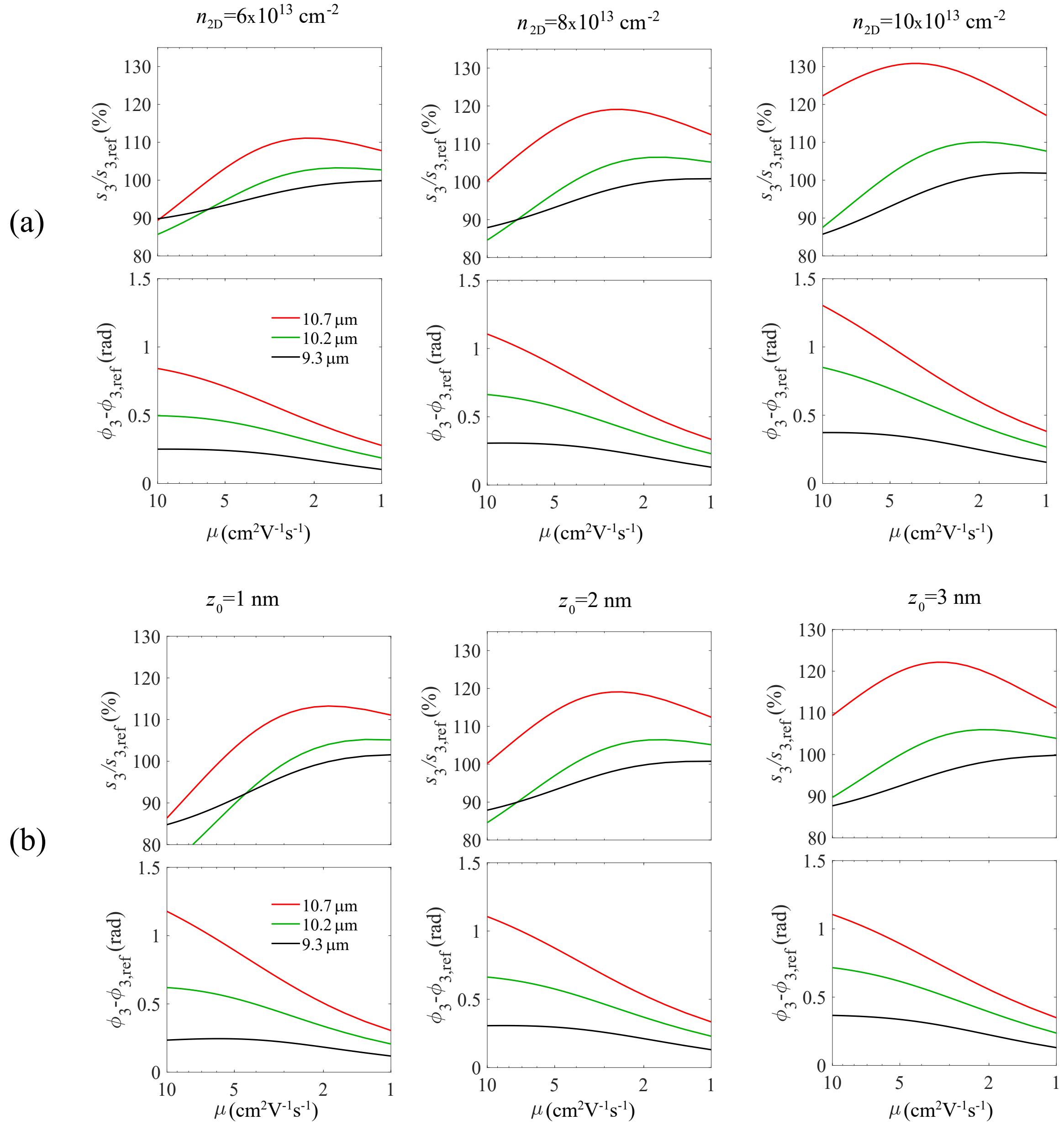

Supplementary Figure 4. Influence of the carrier density n_{2D} (a) and the decay length z_0 (b) on the calculated near-field contrasts.

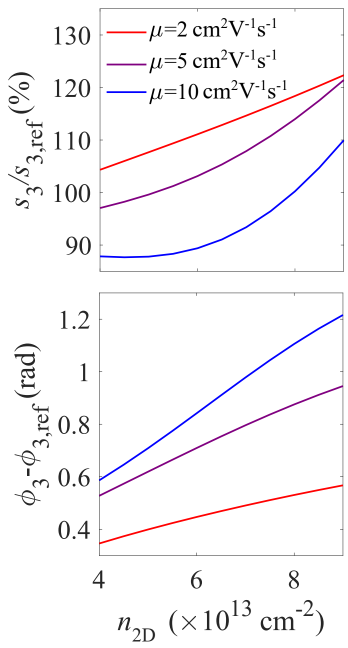

Supplementary Figure 5. Calculated near-field signals as a function of the carrier density for different values of the optical mobility at 10.7 μm.

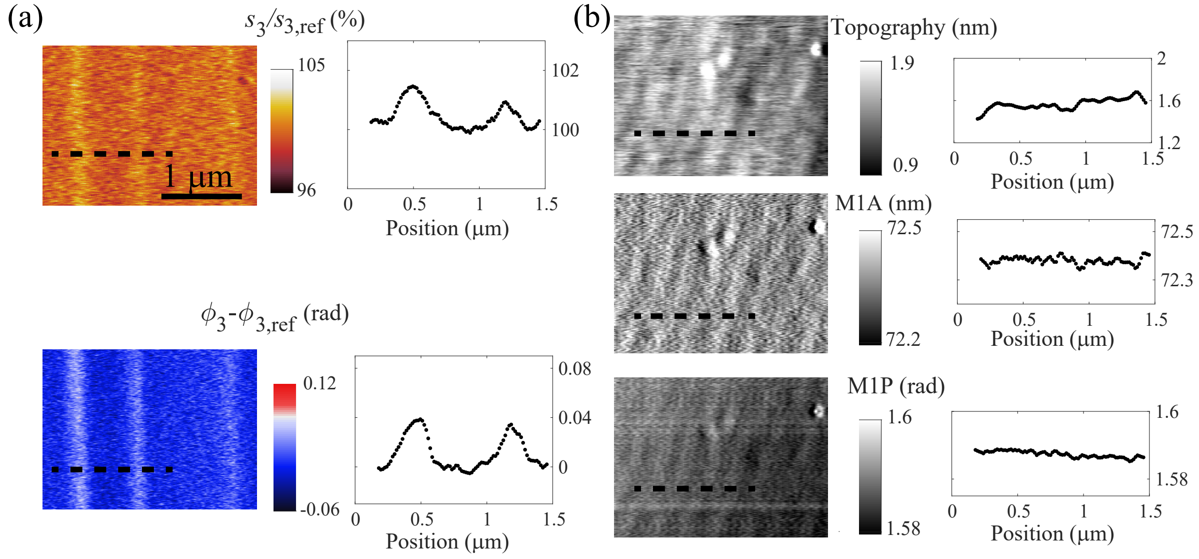

Supplementary Figure 6. Near-field images (a), AFM topography, tapping amplitude and phase (b) measured on the same area, which contains conducting wires written 100 minutes before. Notably, the topography does not show any structure related to the wires. The profile curves are shown for the same positions on the images indicated by the dashed lines.

Supplementary Note 7: Estimation of the width of an AFM-written wire

The width of a conducting channel realized with the AFM-writing method can be estimated using the so-called cutting method. By scanning the (positively) biased AFM tip between two electrodes and monitoring in real-time the total conductance between them, one can observe a sharp increase when tip touches the second electrode[10]. The effect is reversed if the tip is scanned across the channel with a negative bias as the conducting path is cut. The distance over which the conductance decreases when the tip cuts the wire gives an estimation of the channel width. Supplementary Fig. 7 shows the cutting analysis of a wire made using the same writing parameters of our experiment. The curve is fitted using a sigmoidal function and the width is defined as the full width at half maximum of its derivative. A small tip bias (-3 V) applied to the tip during the cut improves the resolution of the technique, however it is not enough to pinch the conducting wire. As a consequence, the total conductance is reduced, but not completely suppressed by the cut.

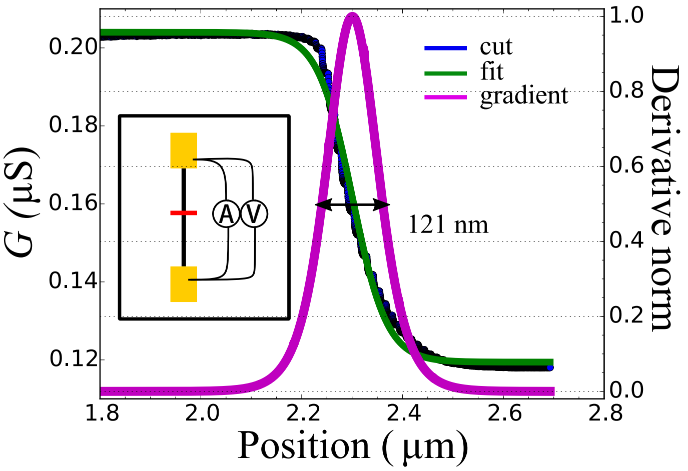

Supplementary Figure 7. Total conductance between two electrodes as a function of the tip position during a cut. The pink curve is the derivative of the experimental curve fit. The wire was written with the same parameters used in our experiment, the cut was performed applying -3V to the tip and moving it at a speed of 30 nm/s. In the inset a sketch of the experiment geometry. A conducting wire (black line) connects two electrodes. The tip trajectory during the cut (x axis in the main figure) is represented by the red line.

[1] Zhang, Z., Choi, B., Flik, M. & Anderson, A. C. Infrared refractive indices of $LaAlO_3$, $LaGaO_3$, and $NdGaO_3$. *J. Opt. Soc. Am. B* **11**, 2252–2257 (1994).

[2] van Mechelen, J. L. M. Charge and Spin electrodynamics of $SrTiO_3$ and $EuTiO_3$ studied by optical spectroscopy. Ph.D. thesis, University of Geneva (2010).

[3] Dubroka, A. *et al.* Dynamical response and confinement of the electrons at the $LaAlO_3/SrTiO_3$ interface. *Phys. Rev. Lett.* **104**, 156807 (2010).

[4] Lewin, M. *et al.* Nanospectroscopy of infrared phonon resonance enables local quantification of electronic properties in doped $SrTiO_3$ ceramics. *Adv. Funct. Mater.* **28**, 1802834 (2018).

[5] Heavens, O. S. *Optical Properties of Thin Films* (Dover, New York, 1965).

[6] Knittl, Z. *Optics of Thin Films: An Optical Multilayer Theory* (Wiley, London, 1976).

[7] Yeh, P. *Optical Waves in Layered Media* (Wiley, New York, 1988).

[8] Fei, Z. *et al.* Infrared nanoscopy of dirac plasmons at the graphene-SiO_2 interface. *Nano Lett.* **11**, 4701–4705 (2011).

[9] Aizpurua, J., Taubner, T., de Abajo, F. J. G., Brehm, M. & Hillenbrand, R. Substrate-enhanced infrared near-field spectroscopy. *Opt. Express* **16**, 1529–1545 (2008).

[10] Boselli, M. *et al.* Characterization of atomic force microscopy written conducting nanowires at $LaAlO_3/SrTiO_3$ interfaces. *Appl. Phys. Lett.* **108**, 061604 (2016).
